# Alfacalcidol improves the growth velocity in children with vitamin D deficiency/insufficiency: A single center retrospective cohort study

**DOI:** 10.1371/journal.pone.0247886

**Published:** 2021-03-08

**Authors:** Satoshi Miyagaki, Mihoko Yamaguchi, Takeshi Ota, Yasuhiro Kawabe, Hidechika Morimoto, Yoshinobu Oka, Jun Mori

**Affiliations:** 1 Department of Pediatrics, Kyoto Prefectural University of Medicine, Graduate School of Medical Science, Kyoto, Japan; 2 Department of Pediatric Orthopaedics, Kyoto Prefectural University of Medicine, Graduate School of Medical Science, Kyoto, Japan; Kobe University Graduate School of Medicine School of Medicine, JAPAN

## Abstract

**Objectives:**

To investigate the growth velocity-improving effects of vitamin D replacement therapy in pediatric patients diagnosed with vitamin D deficiency and insufficiency.

**Study design:**

A retrospective cohort study was conducted in 34 pediatric patients diagnosed with vitamin D deficiency/insufficiency. Based on the clinical findings, the subjects were divided into two groups: a bowed leg (BL) group and a non-bowed leg (non-BL) group. After the initiation of alfacalcidol, the standard deviation score (SDS) of their heights, weights and growth velocities in each group were monitored.

**Results:**

The median age at the first visit was significantly lesser in the BL group (1.58 years old [interquartile range (IQR): 1.33, 2.17]) than that in the non-BL group (3.00 years old [IQR: 2.33, 3.67]). On the contrary, the SDS for height was significantly lower in the non-BL group (-2.27 [IQR: -2.63, -1.94]) than that in the BL group (-1.37 [IQR: -1.91, -1.07]). One-year treatment with alfacalcidol showed significant improvements in both height SDSs and growth velocity SDSs not only in the BL group but also in the non-BL group.

**Conclusions:**

The current study revealed that vitamin D replacement therapy improved the growth rate in children with vitamin D deficiency/insufficiency, regardless of the presence of BL. This study emphasizes the importance of assessing the vitamin D status in children with poor growth rates and suggests that alfacalcidol could be a valid option for the treatment of short stature.

## Introduction

Vitamin D is crucial in maintaining bone and electrolyte metabolic homeostasis [1–5]. Its primary function is to optimize the absorption of Ca and P from the small intestine [6], which promotes normal calcification of the epiphyseal plate and osteoids on the bone surface [7]. Vitamin D is primarily synthesized from provitamin D by the effect of ultraviolet (UV) energy on the skin [1, 5, 6]. Therefore, vitamin D deficiency/insufficiency is a serious problem for the modern population that tends to avoid exposure to UV rays [2, 4, 5]. Particularly in children, vitamin D deficiency/insufficiency impairs bone maturation and causes rickets with symptoms such as lower-limb deformation (e.g., bow-legs and X-legs) and short stature [1, 2, 8, 9].

25-hydroxyvitamin D (25[OH]D) is an intermediate metabolite in the process of formation of vitamin D and is the best indicator of vitamin D sufficiency in the body [1–3]. The cutoff value of serum 25(OH)D level is slightly different in each country, and a consensus has not yet been reached [2–4]. In Japan, those with vitamin D deficiency/insufficiency are diagnosed according to the following criteria: serum 25(OH)D level less than 20 ng/mL is considered as vitamin D deficiency and serum 25(OH)D level less than 30 ng/mL but not less than 20 ng/mL is considered as vitamin D insufficiency [3].

Vitamin D replacement therapy is considered for patients diagnosed with vitamin D deficiency/insufficiency [5, 6, 10, 11]. In Japan, natural vitamin D has not been approved; therefore, active vitamin D, alfacalcidol, is majorly used to treat vitamin D deficiency/insufficiency. An adequate supplementation of vitamin D is expected to normalize serum 25(OH)D levels and improve clinical symptoms by correcting bone metabolism. However, to the best of our knowledge, the patient population in which vitamin D replacement therapy has a greater effect in improving the symptoms is yet to be elucidated. The purpose of this study was to examine the significance of vitamin D supplementation, focusing on whether the presence of bowed legs (BL) affects the improvement of growth velocity.

## Materials and methods

### Ethical consideration

This study was approved by the Clinical Ethics Committee of Kyoto Prefectural University of Medicine (approval number; ERB-C-1601). This study was done with an opt-out method, as it is a retrospective study and, uses a laboratory data and an anthropometric data. The Clinical Ethics Committee approved the opt-out consent. All data was fully anonymized. We posted the opt-out document to provide patients and their parents to refuse to participate in the study by September 30th, 2020 on the website of Department of Pediatrics, Kyoto Prefectural University of Medicine.

### Subjects

The subjects were prepubertal children who visited the Pediatric Endocrinology Division of Kyoto Prefectural University of Medicine due to short stature, growth velocity decline, and/or BL from April 1, 2016 to June 30, 2020. In addition, they were diagnosed with a vitamin D insufficiency, vitamin D deficiency, or vitamin D deficiency rickets through blood and radiographic examination. Pathological varus knee, so called “bowed legs”, was defined as an intercondylar distance ≥ 3 cm. Short stature was defined as ≤ -2 of the standard deviation score (SDS) for the same sex and age. We used the diagnostic criteria established by JSPE. The state of vitamin D insufficiency was defined as 25(OH)D levels ≤ 30 ng/mL, and that of vitamin D deficiency, ≤ 20 ng/mL. The clinical symptoms of rickets included deformities of the lower extremities, gait abnormality, enlarged anterior fontanel, rachitic rosary, swollen joints, pathological fracture, and growth retardation. Radiographic findings of rickets included irregularity, cupping, fraying, and spraying of the metaphysis.

### Methods

At the first visit, the patients were checked for clinical symptoms; additionally, their growth charts and histories were checked. We also obtained details of their family history and the heights of their parents. Accordingly, the target height (TH) was calculated by the following method [12]:

TH for male = (father’s height + mother’s height +13) / 2 (cm)
TH for female = (father’s height + mother’s height -13) / 2 (cm)

Further, blood tests were performed to assess for the levels of Ca, P, alkaline phosphatase (ALP), 25(OH)D, and parathyroid hormone (PTH), and radiography was performed to investigate ricket-related deformities in the limb bones (e.g., the distal ends of the radius and ulna, and the knee joints). Radiographic findings of rickets were evaluated by a pediatric orthopedic surgeon. The patients with a vitamin D insufficiency level were initiated on oral alfacalcidol. Further, we divided the patients into two groups: patients with BL (BL group) and patients without BL (non-BL group). The growth velocity is a value calculated from the measurement of height at two different time points, i.e. the increase in height during a certain period of time. In this study, we calculated patient growth velocity 3, 6 and 12 months after initiation of alfacalcidol, and further calculated the growth velocity SDS with reference to the report by Suwa *et al* [13]. The excluded patients were children with underlying diseases that can cause short stature for example congenital heart diseases, kidney diseases, congenital hypopituitarism, chromosomal abnormalities, and bone system diseases. The above information was collected from the patients’ medical records after which a retrospective study was conducted.

### Statistical analysis

To compare the medians between the two groups, the Mann-Whitney U test was used. Fisher’s exact test was used for testing the relevance of the two-way factors in a contingency table. The mixed model repeated measures (MMRM) analysis was carried out to assess the effect of alfacalcidol on height SDS, weight SDS and height growth velocity SDS at each time point. Models included subjects as a random effect and age, treatment time (0M, 3M, 6M, 12M), the presence or absence of BL and their interaction as fixed effects. Differences with a *p*-value less than 0.05 were regarded as statistically significant. All statistical analyses were performed using SPSS version 26.0 (IBM, Armonk, NY, USA).

## Results

Fifty-five prepubertal pediatric patients were either insufficient or deficient in vitamin D. The patients with underlying diseases that might cause short stature or bone deformities were excluded. Finally, 34 children were registered as target patients for this research. Patient profiles are shown in Table 1. Of the 34 patients, 17 were in the BL group and the remaining 17 were in the non-BL group. The median intercondylar distance of the patients in the BL group was 4.0 cm (IQR: 4.0, 5.0), whereas it was less than 3 cm in all the subjects in the non-BL group. None of the patients had a history of vitamin D supplementation. The median age at the first visit was significantly lower in the BL group. Height and weight SDSs and body mass indices (BMI) were significantly lower in the non-BL group. The subjects with short stature were clearly more common in the non-BL group. The blood test results revealed no significant differences in the serum Ca, P, ALP, 25(OH)D, and intact PTH levels (Table 1). There were no patients with vitamin D- deficient hypocalcemia. Moreover, the rate of vitamin D deficiency/insufficiency did not differ between the groups, but the positivity rate of rickets findings on radiographs was significantly higher in the BL group (Table 2).

**Table 1:** Characteristics of patients enrolled in this study at first consultation.

	BL (n = 17)	non-BL (n = 17)	*p*-value
Demographics and anthropometrics			
Sex (male), n (%)	13 (76)	8 (47)	0.058
Age, years	1.58 (1.33, 2.17)	3.00 (2.33, 3.67)	<0.01
SDS for height	-1.37 (-1.91, -1.07)	-2.27 (-2.63, -1.94)	<0.05
SDS for weight	-0.15 (-0.63, 0.62)	-1.64 (-2.19, -1.44)	<0.01
BMI	17.3 (16.4, 18.4)	15.5 (14.8, 16.4)	<0.01
Short stature, n (%)	4 (23.5)	12 (70.6)	<0.01
Biochemical parameters			
Ca (mg/dl)	10.1 (9.7, 10.2)	9.6 (9.6, 9.9)	0.086
P (mg/dl)	5.2 (5.0, 5.5)	5.1 (4.8, 5.4)	0.300
ALP (IU/L)	875 (655, 1320)	698 (623, 803)	0.163
25(OH)D (ng/ml)	16.0 (13.0, 20.4)	14.2 (11.4, 20.0)	0.959
intact-PTH (pg/ml)	37.5 (32.0, 48.0)	41.5 (31.8, 45.3)	0.763

Data are shown as mean (IQR; 25^th^, 75^th^ percentile).

**Table 2:** The ratios of vitamin D deficiency/insufficiency and rickets findings on X-rays.

	BL	non-BL	*p*-value
	(n = 17)	(n = 17)	
25(OH)D levels			
insufficiency n (%)	5 (29.4)	4 (23.5)	0.5
deficiency, n (%)	12 (70.6)	13 (76.5)	
Rickets findings			
positive, n (%)	17 (100)	10 (58.8)	<0.01
negative, n (%)	0 (0)	7 (41.2)	

**Fig 1:**
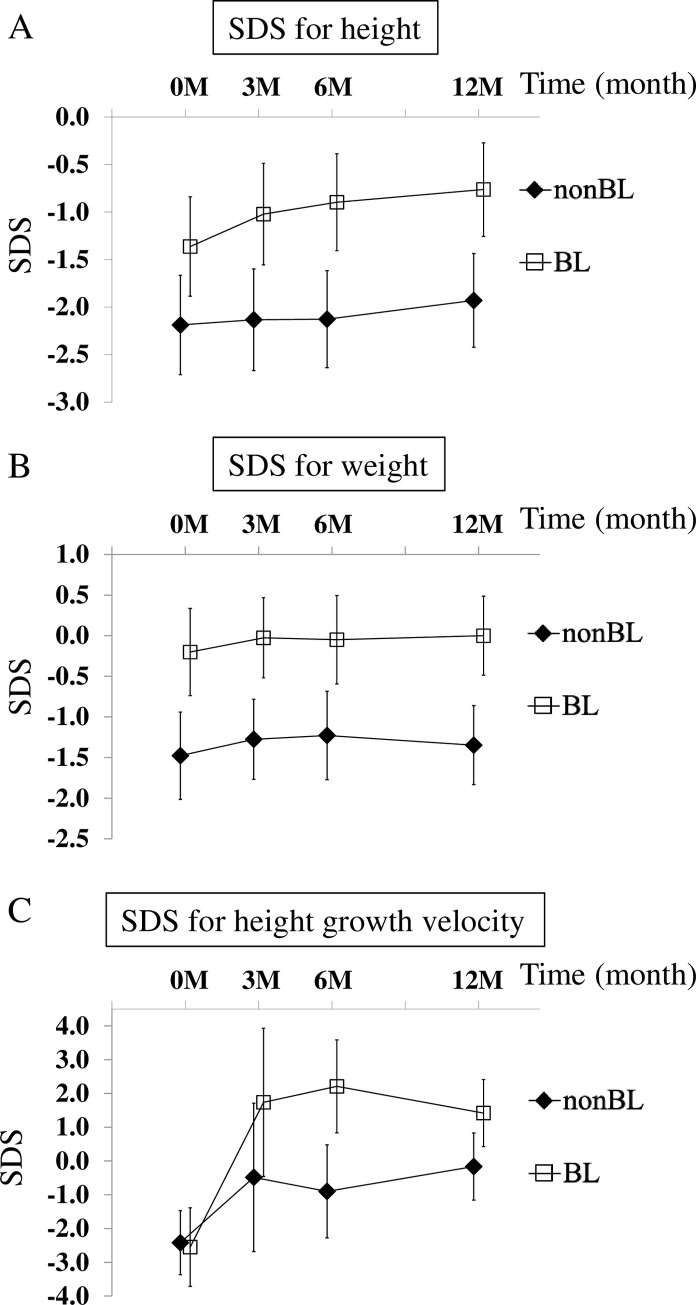
Changes in height SDS (A), weight SDS (B), and height growth velocity SDS (C) during the clinical course. Errors bars represent 95% confidence intervals.

Further, we administered the subjects with alfacalcidol. There was no significant difference in the dosage between the two groups (BL: 0.10 μg/kg/day [IQR: 0.090, 0.117], non-BL: 0.093 μg/kg/day [IQR: 0.080, 0.101] [*p* = 0.168]). During the clinical course, we monitored the urinary calcium/urinary creatinine ratio and adjusted the dose as needed. The patients did not experience any side effects associated with oral alfacalcidol. After starting vitamin D replacement therapy, the heights and weights of the two groups were monitored. Both height and weight SDSs of the BL group were significantly higher than those of the non-BL group throughout the observation period (Fig 1A and 1B). On comparing the height SDS in the same group at the beginning (0 month) and 3 or 6 months after the initiation of vitamin D supplementation, a significant improvement was confirmed only in BL group. At 12 months after the initiation of vitamin D supplementation, a significant improvement was confirmed in both groups after the treatment (*p*<0.001 for BL group, *p* = 0.031 for the non-BL group) (Fig 1A). These results showed vitamin D supplementation improved height SDS regardless of the presence or absence of BL at 12 months after treatment. Regarding body weight, a significant SDS change was found only in the BL group 3 months after the administration of alfacalcidol (Fig 1B).

With respect to the height growth velocity SDSs (Fig 1C), both groups showed a negative value of the height growth rate, and there was no difference between the two groups before the initiation of vitamin D replacement therapy (<0 M). At each treatment time (3M, 6M, 12M), the growth velocity SDS of the BL group had significantly improved compared to before treatment. However, for non-BL group, the height growth velocity SDS only at 12M significantly improved compared to before treatment, even though the value was still negative (*p* = 0.003). Finally, a comparison of before and after intervention of treatment in each group revealed that both groups had significantly improved height growth velocity SDSs one year after the initiation of vitamin D supplementation (*p*<0.001 for BL group, *p* = 0.003 for non-BL group).

## Discussion

As far as we know, few studies have shown the growth velocity-improving effects of oral vitamin D replacement therapy on children with poor growth rates who have vitamin D deficiency/insufficiency [14, 15]. This study included patients with clinical symptoms that could be caused by vitamin D deficiency/insufficiency, such as lower-limb deformation and growth failure. Since all the subjects were residing in the same city (Kyoto, Japan), regional differences in exposure to sunlight hours were considered to have little involvement in their clinical symptoms. The median age of the enrolled subjects in the current study was 2.21 years (IQR: 1.52, 3.00) (Table 3), which was slightly higher than that in previous reports on children with vitamin D deficiency [1, 16–23]. The proportion of BL was approximately the same; however, patients with poor growth (including short stature) accounted for 47% of the total patients in this study, which was more frequent than that in previous studies. A previous study examined the causes of short stature in 70 children and found that growth hormone deficiency was the most common cause (69%), followed by vitamin D deficiency (63%); however, the prevalence of isolated vitamin D deficiency was only 6% [24]. The authors stated that vitamin D deficiency often coexisted with other endocrine disorders and was not a major cause of short stature. On the other hand, in healthy Japanese children aged 0–48 months, 21% (61 out of 290) of the subjects had vitamin D deficiency (25(OH)D value < 20 ng/ml) [2]. However, there was no mention of their heights in this report. The high proportion of short stature in this study is largely influenced by the study design. However, a low rate of vitamin D deficiency does not appear to be a cause of short stature, and pediatricians should be more conscious of serum 25(OH)D levels.

**Table 3:** Summary of previous reports associated with vitamin D deficiency rickets in childhood.

Country,	Number	Age	Bowed legs	Poor growth	25(OH)D	Reference
published year	of cases	(years)	(%)	(%)	(ng/mL)	
Japan, 2002	20	1.82 ± 0.76^a^	60	20	7.7 (4.0–11.0)^b^	[13]
UK, 2004	36	<13	27.8	5.6	2.7 [1.1–5.6]^c^	[14]
Japan, 2005	8	1.32 ± 0.78^a^	75	N.I.	11.6 ± 5.6^a^	[15]
UK, 2006	24	<5	45.8	N.I.	N.L.	[16]
Canada, 2007	104	1.4 ± 0.9^a^	42	5	<11	[17]
Japan, 2009	31	1.4 (0.8–1.65)^b^	48.4	N.I.	N.L.	[18]
Qatar, 2012	45	1.9 ± 0.5^a^	64.4	26.7	<10	[19]
New Zealand, 2015	58	1.4 (0.3–11)^b^	29.3	25.9	4.8 (2.0–19.2)^b^	[20]
Japan, 2018	250	1.75 (1.50–2.33)^b^	83	20	9.0 (6.0–13.8)^b^	[1]
Current study (Japan)	34	2.21 (1.52–3.00)^b^	50	47	15.2 (11.6–19.9)^b^	

N.I., not included; N.L., not listed

^a^, mean ± SD

^b^, median (IQR)

^c^, median [range].

Furthermore, there were no significant differences between the two groups in terms of biochemical test data (Ca, P, ALP, 25(OH)D, and intact-PTH) (Table 2). Unlike previous reports [1, 16, 18, 23], no patients had severe hypocalcemia and hypophosphatemia, and, fortunately, no patients developed convulsions. The population of patients with BL accounted for 50% of the total, but the median vitamin D levels were slightly higher than those in previous reports, suggesting that the severity of vitamin D deficiency might be relatively mild in this study (Table 3).

There was no significant difference in the severity of vitamin D deficiency (Table 2), but the radiological findings of rickets were clearly higher in the BL group. Therefore, the severity of vitamin D deficiency does not appear to be a major factor in determining the development of BL in patients. It may be due to an association between the point in time when low vitamin D levels become apparent and the formation of BL, as the BL group was younger at the first visit than the non-BL group (Table 1). That is, in addition to the bone fragility caused by a vitamin D insufficient state in early infancy, a load on the lower limb bones with the initiation of walking may contribute to the development of BL [7, 8]. On the other hand, if vitamin D deficiency occurs later, BL may be less likely to develop, despite the radiological findings of rickets.

When vitamin D supplementation was performed in the pediatric patients with vitamin D deficiency/insufficiency, the children in the BL group showed significantly better improvement in their height SDSs than those in the non-BL group during the entire observation period (Fig 1A). In fact, in the BL group, the height growth velocity SDS remarkably increased within the first 3 months after treatment initiation (Fig 1C). Therefore, it is suggested that early improvement is crucial for maximizing the effect of improvement of height growth velocity by vitamin D replacement therapy. More interestingly, a significant increase in the height SDS (*p* = 0.031) and height growth velocity SDS (*p* = 0.003) were also seen in the non-BL groups (Fig 1A and 1C). As shown in Table 1, the age at first visit was significantly lower in the BL group than in the non-BL group. However, the MMRM analysis revealed that one year administration of alfacalcidol had a significantly greater effect on improving the growth velocity in both of the BL group and the non-BL group even after adjusting age at the first visit. In 76.5% (13/17) of the patients in the BL group, the pathological varus knee that was present before the treatment initiation disappeared within one year after the induction of oral alfacalcidol. Moreover, the symptoms of the remaining subjects also tended to improve with the treatment. Vitamin D supplementation probably corrected bone fragility and improved lower limb bone deformities, which contributed to the improvements in their height and growth velocities.

At the first visit, the heights SDS as well as the weights SDS were obviously lower in the non-BL group than those in the BL group (Table 1). In addition to vitamin D deficiency, children in the non-BL group were likely to be insufficient in other factors that influence their growth, and it can be expected that their physiques were originally small due to various causes. The median SDS of IGF-1 at the first visit in the non-BL group was -0.93 (IQR: -1.54, -0.46), which was within the standard range. An evaluation of growth hormone secretion ability in 6 cases (1 boy, 5 girls) in the non-BL group returned normal results. Additionally, none of the patients had hypothyroidism. Chromosome G-bandings were performed on 7 out of 9 girls revealed was no involvement of Turner syndrome, one of the causes of short stature in girls. The target height of the non-BL group was 170.8 cm for boys and 153.8 cm for girls, which was within the standard range of adult heights in Japan, and thus, idiopathic short stature (familial short stature) seemed to be less involved. However, other factors influencing their growth were not fully examined. Among the various factors involved in growth, the influence of vitamin D deficiency/insufficiency on height may not be a central factor, but it should not be ignored [24]. Therefore, even in pediatric patients with short stature without apparent lower limb deformities, pediatricians should more aggressively evaluate 25(OH)D levels and consider vitamin D supplementation because vitamin D replacement therapy can have a positive effect on improving their height growth.

One of the major limitations or this study is the lack of nutritional assessment and the investigation whether breastfeeding alone or formula only/mixed feeding in infancy was an important factor involved in vitamin D deficiency in childhood [2, 5, 8]. A previous report showed that, at the age of 0 to 5 months, breastfed infants had significantly higher risks of developing vitamin D deficiencies than those who were fed formula only or were raised by mixed feeding [2]. In addition, the sufficiency of maternal serum 25(OH)D levels is also an important factor [5]. This is because the concentration of 25(OH)D in breast milk, the only natural feeding route for early infants, is influenced by maternal serum 25(OH)D levels [8, 25–28]. Based on the above, the patients in the BL group might be deficient in vitamin D from early infancy due to breastfeeding. In the presence of poor bone mineralization associated with vitamin D deficiency, BL may develop due to the overload at the initiation of walking. On the other hand, in the non-BL group, vitamin D deficiency may not have become apparent in early infancy due to the low proportion of breastfeeding, and bone calcification may have been secured to some extent. It has been suggested that deficiencies of various nutrients, including vitamin D, may appear after the establishment of gait, and may cause clinical symptoms such as short stature and low weight even without BL. Other limitations are as follows: The target height can be helpful when predicting the final height of children, but the height prognosis of both groups could not be compared because the target height data of the BL group was insufficient in this study. Insulin-like growth factor 1 (IGF-1) is often used to indicate general nutritional status [29]. Because of insufficient IGF-1 data in the BL group, we could not compare the nutritional statuses, other than vitamin D supplementation, of both groups as a height-improving factor during the treatment period. In addition, this study is a small number of observational investigations, and it is necessary to conduct many randomized controlled trials.

In conclusion, vitamin D replacement therapy in children with vitamin D deficiency/insufficiency has been shown to improve their height growth velocities. It is important to evaluate their serum 25(OH)D levels in addition to several other nutritional factors while treating patients with growth disorders, and the initiation of vitamin D replacement should be considered in those who have vitamin D deficiency/insufficiency.

## Supporting information

S1 File (XLSX)

## Abbreviations

BL: Bowed leg
non-BL: non-bowed leg
SDS: standard deviation score
IQR: interquartile range
UV: ultraviolet
25(OH)D: 25-hydroxyvitamin D
JSPE: the Japanese Society for Pediatric Endocrinology
TH: target height
ALP: alkaline phosphatase
PTH: parathyroid hormone
BMI: body mas indices

## References

[1] Kubota T, Nakayama H, Kitaoka T, Nakamura Y, Fukumoto S, Fujiwara I, et al. Incidence rate and characteristics of symptomatic vitamin D deficiency in children: a nationwide survey in Japan. Endocr J. 2018;65(6):593–9. 10.1507/endocrj.EJ18-0008 . 29526992

[2] Nakano S, Suzuki M, Minowa K, Hirai S, Takubo N, Sakamoto Y, et al. Current Vitamin D Status in Healthy Japanese Infants and Young Children. J Nutr Sci Vitaminol (Tokyo). 2018;64(2):99–105. 10.3177/jnsv.64.99 . 29710038

[3] Okazaki R, Ozono K, Fukumoto S, Inoue D, Yamauchi M, Minagawa M, et al. Assessment criteria for vitamin D deficiency/insufficiency in Japan: proposal by an expert panel supported by the Research Program of Intractable Diseases, Ministry of Health, Labour and Welfare, Japan, the Japanese Society for Bone and Mineral Research and the Japan Endocrine Society [Opinion]. J Bone Miner Metab. 2017;35(1):1–5. 10.1007/s00774-016-0805-4 . 27882481

[4] Fernandez Bustillo JM, Fernandez Pombo A, Gomez Bahamonde R, Sanmartin Lopez E, Gualillo O. Vitamin D levels in a pediatric population of a primary care centre: a public health problem? BMC Res Notes. 2018;11(1):801. 10.1186/s13104-018-3903-7 . 30409229 PMC6225586

[5] Saggese G, Vierucci F, Boot AM, Czech-Kowalska J, Weber G, Camargo CA Jr., et al. Vitamin D in childhood and adolescence: an expert position statement. Eur J Pediatr. 2015;174(5):565–76. 10.1007/s00431-015-2524-6 . 25833762

[6] Antonucci R, Locci C, Clemente MG, Chicconi E, Antonucci L. Vitamin D deficiency in childhood: old lessons and current challenges. J Pediatr Endocrinol Metab. 2018;31(3):247–60. 10.1515/jpem-2017-0391 . 29397388

[7] Pettifor JM, Prentice A. The role of vitamin D in paediatric bone health. Best Pract Res Clin Endocrinol Metab. 2011;25(4):573–84. 10.1016/j.beem.2011.06.010 . 21872799

[8] Winzenberg T, Jones G. Vitamin D and bone health in childhood and adolescence. Calcif Tissue Int. 2013;92(2):140–50. 10.1007/s00223-012-9615-4 . 22710658

[9] Ozono K. Today’s nutritional deficiencies- Vitamin D deficiency. Vitamins (Japan). 2012; 86: 28–31.

[10] Zerofsky M, Ryder M, Bhatia S, Stephensen CB, King J, Fung EB. Effects of early vitamin D deficiency rickets on bone and dental health, growth and immunity. Matern Child Nutr. 2016;12(4):898–907. 10.1111/mcn.12187 . 25850574 PMC4610869

[11] Bueno AL, Czepielewski MA, Raimundo FV. Calcium and vitamin D intake and biochemical tests in short-stature children and adolescents. Eur J Clin Nutr. 2010;64(11):1296–301. 10.1038/ejcn.2010.156 . 20736967

[12] Ogata T, Tanaka T, Kagami M. Target height and target range for Japanese children: revisited. Clin Pediatr Endocrinol. 2007;16(4):85–7. 10.1297/cpe.16.85 . 24790351 PMC4004886

[13] Suwa S. Standards for growth and growth velocity in Turner’s syndrome. Acta Paediatr Jpn. 1992;34(2):206–20; discussion 21. 10.1111/j.1442-200x.1992.tb00951.x . 1621526

[14] Rajah J, Jubeh JA, Haq A, Shalash A, Parsons H. Nutritional rickets and z scores for height in the United Arab Emirates: to D or not to D? Pediatr Int. 2008;50(4):424–8. 10.1111/j.1442-200X.2008.02700.x . 18937749

[15] Ganmaa D, Stuart JJ, Sumberzul N, Ninjin B, Giovannucci E, Kleinman K, et al. Vitamin D supplementation and growth in urban Mongol school children: Results from two randomized clinical trials. PLoS One. 2017;12(5):e0175237. 10.1371/journal.pone.0175237 . 28481882 PMC5421751

[16] Nishikura K, Kano K, Arisaka O, Morishima N. Case of incidentally diagnosed vitamin D deficiency rickets: a review of literature from Japan and a proposal for reintroduction of vitamin D2. Pediatr Int. 2002;44(2):179–82. 10.1046/j.1328-8067.2001.01514.x . 11896880

[17] Ladhani S, Srinivasan L, Buchanan C, Allgrove J. Presentation of vitamin D deficiency. Arch Dis Child. 2004;89(8):781–4. 10.1136/adc.2003.031385 . 15269083 PMC1720051

[18] Miyako K, Kinjo S, Kohno H. Vitamin D deficiency rickets caused by improper lifestyle in Japanese children. Pediatr Int. 2005;47(2):142–6. 10.1111/j.1442-200x.2005.02041.x . 15771690

[19] Callaghan AL, Moy RJ, Booth IW, Debelle G, Shaw NJ. Incidence of symptomatic vitamin D deficiency. Arch Dis Child. 2006;91(7):606–7. 10.1136/adc.2006.095075 . 16595644 PMC2082851

[20] Ward LM, Gaboury I, Ladhani M, Zlotkin S. Vitamin D-deficiency rickets among children in Canada. CMAJ. 2007;177(2):161–6. 10.1503/cmaj.061377 . 17600035 PMC1913133

[21] Matsuo K, Mukai T, Suzuki S, Fujieda K. Prevalence and risk factors of vitamin D deficiency rickets in Hokkaido, Japan. Pediatr Int. 2009;51(4):559–62. 10.1111/j.1442-200X.2009.02834.x . 19419526

[22] Soliman A, De Sanctis V, Adel A, El Awwa A, Bedair S. Clinical, biochemical and radiological manifestations of severe vitamin d deficiency in adolescents versus children: response to therapy. Georgian Med News. 2012;(210):58–64. . 23045422

[23] Wheeler BJ, Dickson NP, Houghton LA, Ward LM, Taylor BJ. Incidence and characteristics of vitamin D deficiency rickets in New Zealand children: a New Zealand Paediatric Surveillance Unit study. Aust N Z J Public Health. 2015;39(4):380–3. 10.1111/1753-6405.12390 26122859

[24] Jawa A, Riaz SH, Khan Assir MZ, Afreen B, Riaz A, Akram J. Causes of short stature in Pakistani children found at an Endocrine Center. Pak J Med Sci. 2016;32(6):1321–5. 10.12669/pjms.326.11077 . 28083018 PMC5216274

[25] Taylor SN, Wagner CL, Hollis BW. Vitamin D supplementation during lactation to support infant and mother. J Am Coll Nutr. 2008;27(6):690–701. 10.1080/07315724.2008.10719746 . 19155428

[26] Brannon PM, Picciano MF. Vitamin D in pregnancy and lactation in humans. Annu Rev Nutr. 2011;31:89–115. 10.1146/annurev.nutr.012809.104807 . 21756132

[27] Aghajafari F, Nagulesapillai T, Ronksley PE, Tough SC, O’Beirne M, Rabi DM. Association between maternal serum 25-hydroxyvitamin D level and pregnancy and neonatal outcomes: systematic review and meta-analysis of observational studies. BMJ. 2013;346:f1169. 10.1136/bmj.f1169 . 23533188

[28] Harvey NC, Holroyd C, Ntani G, Javaid K, Cooper P, Moon R, et al. Vitamin D supplementation in pregnancy: a systematic review. Health Technol Assess. 2014;18(45):1–190. 10.3310/hta18450 . 25025896 PMC4124722

[29] Caregaro L, Favaro A, Santonastaso P, Alberino F, Di Pascoli L, Nardi M, et al. Insulin-like growth factor 1 (IGF-1), a nutritional marker in patients with eating disorders. Clin Nutr. 2001;20(3):251–7. 10.1054/clnu.2001.0397 . 11407872

